# Digital auscultation as a diagnostic aid to detect childhood pneumonia: A systematic review

**DOI:** 10.7189/jogh.12.04033

**Published:** 2022-04-23

**Authors:** Salahuddin Ahmed, Saima Sultana, Ahad M Khan, Mohammad S Islam, GM Monsur Habib, Ian M McLane, Eric D McCollum, Abdullah H Baqui, Steven Cunningham, Harish Nair

**Affiliations:** 1 Usher Institute, University of Edinburgh, Edinburgh, UK; 2 Projahnmo Research Foundation, Dhaka, Bangladesh; 3 Child Health Research Foundation, Dhaka, Bangladesh; 4 Bangladesh Primary Care Respiratory Society, Khulna, Bangladesh; 5 Sonavi Labs, Baltimore, Maryland, USA; 6 Global Program for Pediatric Respiratory Sciences, Eudowood Division of Paediatric Respiratory Sciences, School of Medicine, Johns Hopkins University, Baltimore, Maryland, USA; 7 Department of International Health, Bloomberg School of Public Health, Johns Hopkins University, Baltimore, Maryland, USA; 8 Department of Child Life and Health, Centre for Inflammation Research, University of Edinburgh, Edinburgh, UK

## Abstract

**Background:**

Frontline health care workers use World Health Organization Integrated Management of Childhood Illnesses (IMCI) guidelines for child pneumonia care in low-resource settings. IMCI guideline pneumonia diagnostic criterion performs with low specificity, resulting in antibiotic overtreatment. Digital auscultation with automated lung sound analysis may improve the diagnostic performance of IMCI pneumonia guidelines. This systematic review aims to summarize the evidence on detecting adventitious lung sounds by digital auscultation with automated analysis compared to reference physician acoustic analysis for child pneumonia diagnosis.

**Methods:**

In this review, articles were searched from MEDLINE, Embase, CINAHL Plus, Web of Science, Global Health, IEEExplore database, Scopus, and the ClinicalTrial.gov databases from the inception of each database to October 27, 2021, and reference lists of selected studies and relevant review articles were searched manually. Studies reporting diagnostic performance of digital auscultation and/or computerized lung sound analysis compared against physicians’ acoustic analysis for pneumonia diagnosis in children under the age of 5 were eligible for this systematic review. Retrieved citations were screened and eligible studies were included for extraction. Risk of bias was assessed using the Quality Assessment of Diagnostic Accuracy Studies-2 (QUADAS-2) tool. All these steps were independently performed by two authors and disagreements between the reviewers were resolved through discussion with an arbiter. Narrative data synthesis was performed.

**Results:**

A total of 3801 citations were screened and 46 full-text articles were assessed. 10 studies met the inclusion criteria. Half of the studies used a publicly available respiratory sound database to evaluate their proposed work. Reported methodologies/approaches and performance metrics for classifying adventitious lung sounds varied widely across the included studies. All included studies except one reported overall diagnostic performance of the digital auscultation/computerised sound analysis to distinguish adventitious lung sounds, irrespective of the disease condition or age of the participants. The reported accuracies for classifying adventitious lung sounds in the included studies varied from 66.3% to 100%. However, it remained unclear to what extent these results would be applicable for classifying adventitious lung sounds in children with pneumonia.

**Conclusions:**

This systematic review found very limited evidence on the diagnostic performance of digital auscultation to diagnose pneumonia in children. Well-designed studies and robust reporting are required to evaluate the accuracy of digital auscultation in the paediatric population.

Pneumonia is the leading cause of mortality among infectious diseases in children under the age of five globally [1], accounting for an estimated 800 000 deaths per year, more than half of which occur in just five low- and middle-income countries (LMIC) [1,2]. In 2015, the estimated annual incidence of pneumonia in children under five years of age in developing countries was 231 episodes per 1000 children, resulting in about 138 million episodes of clinical pneumonia in this age group [3]. Prompt recognition of illness and care-seeking is critical to reducing pneumonia-related child deaths [4].

Currently, health care providers in LMICs use practical, standardized case management guidelines called the Integrated Management of Childhood Illnesses (IMCI) guidelines developed by the World Health Organization (WHO) for childhood pneumonia care [5,6]. IMCI guidelines have proven to be one of the most important childhood pneumonia interventions for LMICs to date [7,8].

IMCI guidelines prioritize sensitivity over specificity to ensure antibiotic treatment for children with an acute respiratory illness and suspected bacterial pneumonia [6]. Where successfully implemented, this algorithm has shown a 30%-40% reduction in case mortality [8]. Yet, IMCI has moderate sensitivity and a specificity largely contingent on disease severity. Specificity may range from 16% for children with an acute respiratory illness characterized by wheeze [9], to 49% for IMCI-defined non-severe pneumonia (ie, disease lacking clinical danger signs), to 95% for IMCI-defined very severe pneumonia (ie, disease with clinical danger signs) [10]. Limitations of IMCI specificity are thought to be related to the attribution of milder disease to viral pathogens, an epidemiological pattern catalysed by the expanded exposure of children in LMICs to *Haemophilus influenzae* type B and access to pneumococcal conjugate vaccines [11-13]. As a result, while the guidelines ensure few children with true bacterial pneumonia will be overlooked, most children, especially those with a milder illness, receive antibiotics inappropriately, resulting in antibiotic overuse.

Auscultation, the process of listening to the human body’s internal sounds by using a stethoscope [14], has been an effective tool for diagnosing pulmonary diseases for more than two centuries. This requires highly trained health professional, limiting its utility at first-level facilities in LMICs staffed by non-physician health workers. IMCI guidelines do not include lung auscultation in their pneumonia definition for frontline health care workers [6]. The guidelines’ exclusion of auscultation likely results from its high inter-observer variability and subjectivity, regardless of health care providers’ training level [15-19]. Furthermore, traditional stethoscopes are functionally limited since they attenuate higher frequency sounds, like wheezing and crackles, yet transmit ambient noises and tubular resonance effects [17,20]. Digital auscultation may overcome these limitations [21]. Digital auscultation has the potential advantages of signal amplification and ambient noise reduction [22-24], reducing inter-observer reporting variation [25], and not requiring auscultation training for health care providers. Digital stethoscopes can record a patient’s respiratory sounds for automated computerised lung sound analysis, which has been the recent focus of a significant amount of research, with some commercial systems already available [26,27]. These advantages may be important in LMICs as they rely on varying cadres of non-physician clinicians, and as the burden of lower respiratory infections among children in LMICs is high.

To the best of our knowledge, there is currently no systematic review reporting the diagnostic accuracy of digital auscultation to detect adventitious lung sounds in childhood pneumonia. Therefore, we aimed to conduct a systematic review to summarize the current evidence on the diagnostic performance of digital auscultation to identify adventitious lung sounds compared to the physician’s acoustic analysis to diagnose pneumonia in children.

## METHODS

This systematic review was reported according to the Preferred Reporting Items for Systematic Reviews and Meta-Analyses of Diagnostic Test Accuracy (PRISMA-DTA) criteria [28]. The review protocol was registered with the PROSPERO database (registration number CRD42020180821).

### Information sources and search strategy

Search terms (“pneumonia”, “lower respiratory infection”, “auscultation”, “respiratory sound”, “lung sound”, “digital”, “electronic”, “automatic”, “computerized”, “crackles”, “wheeze”, “child”) were used to generate comprehensive search strategies for the following electronic databases: MEDLINE, Embase, Cumulative Index to Nursing and Allied Health Literature (CINAHL) Plus, Web of Science, Global Health, IEEExplore database, Scopus, and ClinicalTrials.gov (http://clinicaltrials.gov/). Search strategies for all databases are shown in Appendix S1 in the **Online Supplementary Document**. The initial search was conducted in February 2019 and was updated on October 27, 2021, with no language restrictions. To avoid missing relevant studies, reference lists of identified studies and relevant reviews were also screened.

### Eligibility criteria

Studies were selected based on the following inclusion criteria:

1. Participants: Children under five years of age.
2. Index test: Digital auscultation/computerized analysis of lung sounds.
3. Reference standards: Physicians’ diagnosis of adventitious lung sounds (crackles and/or wheeze) by conventional auscultation/acoustic analysis of recorded lung sounds.
4. Target condition: Pneumonia.
5. Outcome: Reported diagnostic accuracy measures of the digital/computerized analysis of adventitious lung sounds.
6. Types of studies: Observational and experimental studies.

Studies were excluded if 1) digital auscultation or computerized analysis of lung sounds were not used in the study, 2) reference standard was not a human classification of lung sounds, 3) the reports were reviews, conference proceedings, abstracts, case reports, editorials, and commentaries.

### Screening and selection of studies

Search results were imported and merged into Endnote X9, and duplicates were removed. Two review authors (SA and MSI/SS/GMMH) independently screened the titles and abstracts of all included citations as per the predefined eligibility criteria, followed by a full-text review of all selected articles (SA and SS/AMK). Any discrepancies were resolved through discussion, or an arbiter (HN) was consulted, where necessary. Reasons for exclusion of studies were documented (Appendix S2 in the **Online Supplementary Document**).

### Data extraction and quality assessment

Data extractions were independently carried out by two review authors (SA and AMK) using a standardized pretested data collection template (Appendix S3 in the **Online Supplementary Document**). Any discrepant judgments were evaluated by an arbiter (HN).

Two reviewers (SA and AMK) independently assessed the risk of bias and the applicability of the included studies using the Quality Assessment of Diagnostic Accuracy Studies (QUADAS-2) tool [29]. Non-consensus between the reviewers were resolved through consultation with an arbiter (HN). This tool includes four domains to judge bias in the included study: patient selection, index test, reference standard, and flow and timing. A study would have an overall judgment of “low risk of bias” if it was judged as “low” on all domains. In contrast, it would be judged as a “high risk of bias” if it was judged “high” in one or more domains. The “unclear risk of bias” was categorised only when insufficient data were reported. To judge the concerns regarding the applicability of the included study to the review question, three domains (ie, patient selection, index test, and reference standard) were used.

### Data synthesis

A descriptive synthesis was performed following the predefined review objectives and outcome measures. Meta-analyses could not be performed due to insufficient data and the heterogeneity in included studies in terms of methodology and outcome measures.

## RESULTS

**Figure 1:**
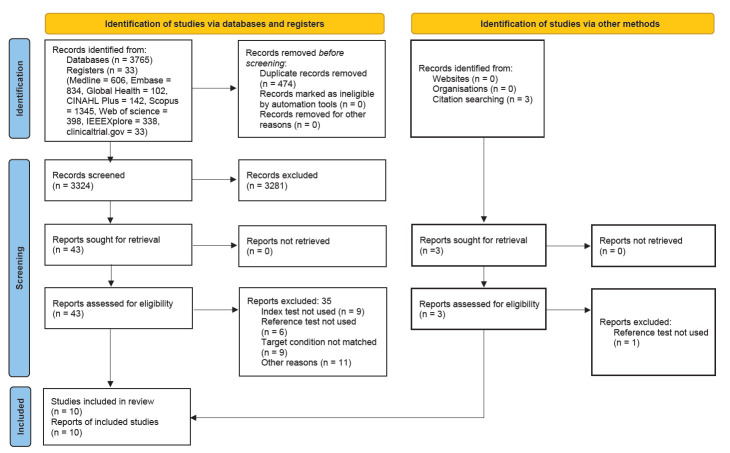
PRISMA flow diagram.

The review process is summarised in **Figure 1** using the PRISMA flow diagram [30]. A total of 3798 citations were identified through the database search. After duplicates were removed, 3324 unique citations remained. In total, 3281 citations were excluded during the title and abstract screening, and 46 full-text articles (including three from citation searching) were reviewed. Of these, 10 articles were eligible for inclusion [21,31-39].

### Characteristics of the included studies

**Table 1** summarises the characteristics of the included studies. Of the selected articles, five studies evaluated lung sound recordings from primary studies, among which one study was conducted across multiple centres in Africa (The Gambia, Kenya, South Africa, Zambia) and Asia (Bangladesh, Thailand) [21], one in Australia [36], one in India [37], and the remaining two were from the same study conducted in Egypt [31,35]. These five studies recruited children from different age groups and varied from one another in the number of study subjects. For instance, one study enrolled 1157 children aged 1-59 months [21], one study enrolled 600 children aged 0-12 years [31,35], while another study had only 20 children aged 4.6-17.1 years [36]. However, one study recruited 256 children but did not report any information on the participants’ age [37]. Of these five studies, one study obtained lung sound recordings in outpatient and busy clinical settings [21], while another study recorded the lung sounds in a quiet room in the hospital [36]. Three studies also recorded lung sounds in a hospital setting [31,35,37], but is unclear whether they were recorded in a controlled environment or a noisy clinical setting. The other five studies [32-34,38,39] evaluated their proposed work using the publicly available International Conference on Biomedical and Health Informatics (ICBHI) scientific challenge respiratory sound database containing 920 annotated lung sound recordings from 126 subjects (49 children and 77 adults) [40,41].

**Table 1:** Study characteristics

Author, year	Country	Study type	Population	Number of subjects	Clinical condition of the subjects	Sound/ pathology analyzed	Number of recordings studied	Lung sound recording device	Feature extraction method	Sound classification method	Outcome/Result
Fasseeh et al. 2015	Egypt	Case-control	Infants and children (0-12 y)	Case = 500 Control = 100	Not reported	Wheeze, stridor, rattle, normal	592	3M Littmann Electronic Stethoscope 3200	Short-time Fourier Transform (STFT)	Dynamic time warping (DTW) algorithm	Reported accuracy of validation for all wheezes was 81.93% (<12 months = 82.81% & ≥12 months = 89.15%). Reported accuracy of validation for all normal sounds was 89% (<12 months = 96.15% & ≥12 months = 90.54%).
Khan et al. 2017	India		Children	254	Not reported	Normal and pathological	254	Littmann 3200 electronic stethoscope	Short time Fourier transform (STFT)	k- Nearest Neighbour (k-NN); Support Vector Machine (SVM)	k-NN obtained sensitivity, specificity, and accuracy of 90.9%, 92.2% and 91.6%, respectively. SVM obtained sensitivity, specificity, and accuracy of 92.2%.
Kevat et al. 2017	Australia		Children (median age = 6.7 y)	20	Cystic fibrosis, lower respiratory tract infection, asthma, preschool wheeze	Wheeze, crackles, normal	156	Littmann 3200 Electronic Stethoscope; Clinicloud DS	Not reported	Audio spectrographic analysis	Concordance between the Littmann electronic stethoscope and standard auscultation was found to be moderate for wheeze (κ = 0.44) and almost perfect for crackles (κ = 1.0). Concordance between the Clinicloud DS and standard auscultation was found to be moderate for wheeze (κ = 0.55) and almost perfect for crackles (κ = 1.0).
Abougabal et al. 2018	Egypt	Analysis of recorder lung sounds	Infants and Children (<13 years)	600	Not reported	Stridor, rattle and wheeze, normal	592	3M Littmann Electronic Stethoscope 3200	Wavelet Transform (WT) coefficients	Dynamic time warping (DTW) algorithm	Reported recognition accuracy of 88.2% for wheeze and 86% for normal breath sounds.
Emmanouilidou et al. 2018	Gambia, Kenya, South Africa, Zambia, Bangladesh, Thailand	Case-control	Children (median age 7 ± 11.4 months)	1157	Pneumonia, Normal	Normal, abnormal (wheeze and/or crackle)	1095	ThinkLabs ds32a	Rich spectro-temporal feature space	Support-Vector Machine (SVM) classifier	The classification system achieved an accuracy of 86.7%, sensitivity of 86.9%, and specificity of 86.6%
Chen et al. 2019		Analysis of respiratory sound database*	Not mentioned	Not mentioned	Not mentioned	Wheeze, crackle, normal	489	3M Littmann 3200 Electronic Stethoscope; Welch Allyn Elite Meditron	Optimized-S- Transform (OST)	Deep Residual Networks (ResNets)	Classification accuracy of 98.79% with sensitivity of 96.27% and specificity of 100% was obtained to classify wheeze, crackle, and normal sounds.
Perna et al. 2019		Analysis of respiratory sound database*	Children, adults	126	Pneumonia, Bronchiectasis, Bronchiolitis, COPD, Healthy, URTI	Normal, wheeze, crackle, both	920	AKG C417 L Microphone; 3M Littmann Classic II SE. Stethoscope; 3M Littmann 3200 Electronic Stethoscope; Welch Allyn Meditron Master Elite Electronic Stethoscope	Mel-Frequency Cepstral Coefficients (MFCC)	Recurrent Neural Networks (RNN) models: Long Short-Term Memory (LSTM) and Gated Recurrent Unit (GRU)	A sensitivity of 0.62 and specificity of 0.85 were reported for LSTM based model on four class anomaly driven prediction. (i.e., normal, presence of crackles, presence of wheezes, presence of both)
Acharya et al. 2020		Analysis of respiratory sound database*	Children, adults	126	Pneumonia, Bronchiectasis, Bronchiolitis, COPD, Healthy, URTI	Normal, wheeze, crackle, both	920	AKG C417 L Microphone; 3M Littmann Classic II SE. Stethoscope; 3M Littmann 3200 Electronic Stethoscope; Welch Allyn Meditron Master Elite Electronic Stethoscope	Mel-spectrograms	Hybrid CNN-RNN model	A score of 66.31% was obtained on four class respiratory cycle classification and a score of a 71.81% was obtained for leave-one-out validation.
Alva Alicia et al. 2021		Analysis of respiratory sound database*	Children, adults	126	Pneumonia, Bronchiectasis, Bronchiolitis, COPD, Healthy, URTI	Normal, abnormal	920	AKG C417L Microphone; 3M Littmann Classic II SE. Stethoscope; Littmann 3200 Electronic Stethoscope; Welch Allyn Meditron Master Elite Electronic Stethoscope	Mel-spectogram; Short time Fourier transform (STFT); Mel-Frequency Cepstral Coefficients (MFCC)	Convolutional Neural (CNN) Network Models	Accuracy values of 0.998 and 1 were obtained for normal sounds and abnormal sounds respectively. Accuracy values of 0.9959 and 0.9885 were reported for classification of pneumonia and other diseases.
Shuvo et al. 2021		Analysis of respiratory sound database*	Children, adults	87	Pneumonia, Bronchiectasis, Bronchiolitis, COPD, Healthy, URTI	Chronic classification (healthy, chronic diseases, non-chronic diseases) Pathological classification (Healthy, Bronchiectasis, Bronchiolitis, COPD, Pneumonia, URTI)	917	AKG C417L Microphone; 3M Littmann Classic II SE. Stethoscope; Littmann 3200 Electronic Stethoscope; Welch Allyn Meditron Master Elite Electronic Stethoscope	Hybrid scalogram using empirical mode decomposition (EMD) & continuous wavelet transform (CWT)	Lightweight Convolutional neural network (CNN) model	Weighted accuracy scores of 98.92% for three-class chronic classification and 98.70% for six-class pathological classification were obtained.

*The study analysed International Conference on Biomedical Health Informatics (ICBHI) 2017 data set.

We found only one study that specifically recruited children with IMCI-defined severe or very severe pneumonia and age-matched controls without clinical pneumonia [21], while seven studies included subjects with a variety of respiratory diseases, including pneumonia [32-34,36-39]. The other two studies did not report on the disease condition of the study subjects [31,35,37].

### Stethoscopes used

For studies using the ICBHI data set, lung sounds were recorded using different devices (3M Littmann Classic II SE stethoscope, 3M Littmann 3200 electronic stethoscope, WelchAllyn Meditron Master Elite electronic stethoscope, and AKG C417 L Microphone) [32-34,38,39] from both clinical and home settings [41]. In the other four studies, a 3M Littmann 3200 electronic stethoscope was used [31,35-37], while the Clinicloud DS was also used in one of the studies [36]. The ThinkLabs ds32a digital stethoscope with a microphone (Sony-ICD-UX71-81) affixed to the stethoscope to record ambient noises was used in one study [21].

### Lung sounds classification used

Variations were observed in classifying the lung sounds. For instance, three studies specifically performed the computerized analysis of lung sounds to classify normal and adventitious/pathological sounds [21,33,37], two studies performed a four-class classification (normal, crackle, wheeze, both wheeze and crackle) [32,38], one study performed a three-class classification (normal, crackle, and wheeze) [34], one study classified wheeze and crackles [36] and another two analysed wheeze, rattle, stridor, and normal lung sounds [31,35]. One study performed a three-class chronic classification (healthy, non-chronic diseases, chronic diseases) and six-class pathological classification (healthy, bronchiectasis, bronchiolitis, COPD, Pneumonia, URTI) based on the respiratory sound signal processing and computerised analysis [39].

### Algorithm used to classify lung sounds

A substantial variation was observed in feature extraction methods (ie, the process of identifying distinctive features of respiratory sound signals) and sound classification algorithms/models used in the included studies. For feature extraction, three studies employed Short-time Fourier Transform (STFT) [33,35,37], two studies employed Melspectrogram [32,33], two used Mel Frequency Cepstral Coefficients (MFCC) [33,38], while the other four studies employed enhanced distinctive feature extraction methods –Wavelet Transform (WT) coefficients [31], rich spectro-temporal feature space [21], Optimized-S-Transform (OST) [34], and hybrid scalogram using empirical mode decomposition (EMD) and continuous wavelet transform (CWT) [39]. Wide-ranging sound classification models/algorithms used across the studies are: Dynamic time warping (DTW) algorithm [31,35], Support Vector Machine (SVM) [21,37], k-Nearest Neighbour (k-NN) based classifier [37], audio spectrographic analysis [36], Deep Residual Networks (ResNets) [34], Recurrent Neural Networks (RNN) models with Long Short-Term Memory (LSTM) and Gated Recurrent Unit (GRU) [38], hybrid CNN-RNN model [32], Convolutional Neural Network (CNN) models [33], and lightweight convolutional neural network (CNN) model [39].

### Diagnostic performance of used algorithms/models

Regarding diagnostic performance, diverse methodologies and diagnostic accuracy measures were used. Furthermore, in studies that included participants with different target conditions and age groups (ie, children and adults), the reported performance measures were only limited to overall accuracy on adventitious lung sounds classification [32-34,38] or by pathological conditions classification [39]. For these studies, reported accuracies varied from 66.3% to 100%. Of the five studies that recruited children [21,31,35-37], only one study [21] reported on the accuracy of the computerized sound classification system in children aged 1-59 months in pneumonia. In that study, the accuracy of the classification system was 86.7% (sensitivity = 86.8%; specificity = 86.6%) to differentiate between normal and adventitious lung sounds. Further, the researchers suggested that the system performance varied with the different window analyses of breath cycles ranging from shorter to longer duration. For example, setting the analysis window at 0.5s yielded an accuracy of 84.1% (sensitivity = 87.2%; specificity = 81%) while it was about 77% in longer time windows (>1s); thus, a short window size was recommended [21].

### Assessment of risk of bias and applicability

**Figure 2:**
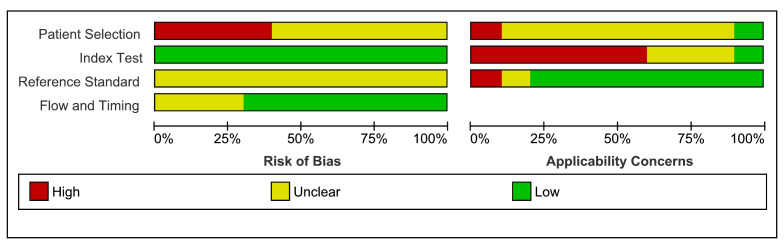
Risk of bias and applicability concerns graph: review authors' judgements about each domain presented as percentages across included studies.

**Figure 3:**
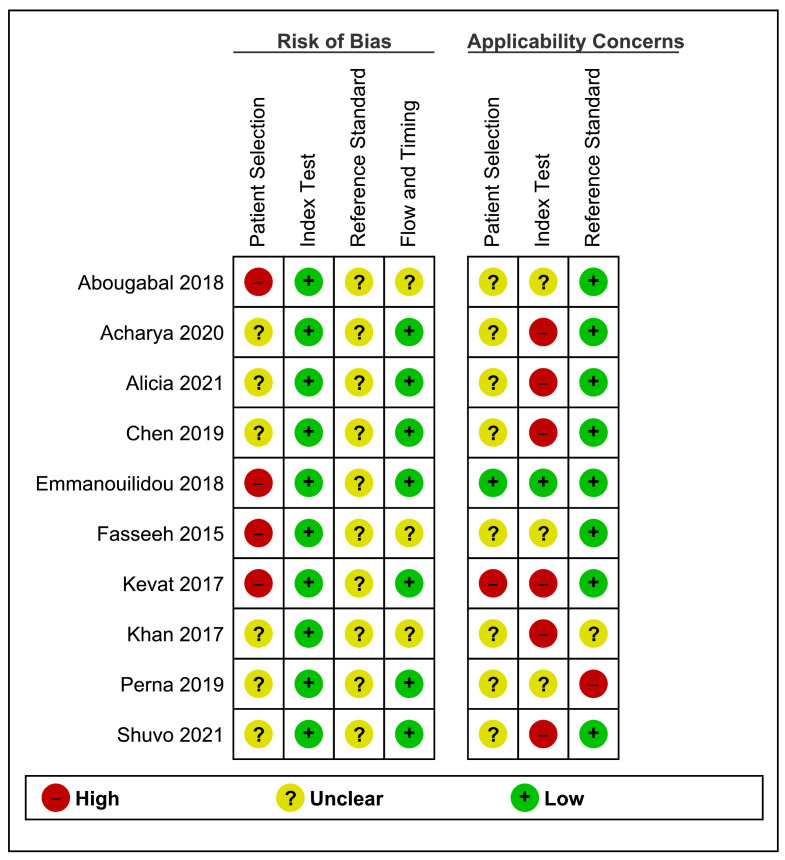
Risk of bias and applicability concerns summary: review authors' judgements about each domain for each included study.

The quality of the included studies according to the QUADAS-2 tool is summarized and displayed graphically in **Figure 2** and **Figure 3**. In general, very few of the included studies met most of the quality criteria. Most of the studies were judged to be of unclear methodological quality because of insufficient reporting. For patient selection, four studies were evaluated as having a high risk of bias, and six were evaluated as having an unclear risk of bias due to inappropriate or poorly described sampling methods. For the index test, all 10 studies were judged as having low risk of bias because there was no chance of non-blinding, as index tests were the machine classifications. For the reference standard, all the studies were evaluated as having an unclear risk of bias due to poor reporting of target conditions and/or blinding status. In the flow and timing domain assessment, seven studies were deemed to have a low risk of bias as the recorded sounds were the second interpretation, and three were evaluated as an unclear risk because of underreporting. The overall concerns regarding applicability for this review were unclear, high, and low, for patient selection, index test, and reference standard, respectively.

## DISCUSSION

This systematic review included studies evaluating the diagnostic performance of digital auscultation/computerized analysis of adventitious lung sounds in pneumonia in children aged under 5 years. The literature search identified 10 articles that met the inclusion criteria. Of these included articles, five of them evaluated lung sound recordings from primary studies, while the remaining five utilised a publicly available respiratory sound database to evaluate their proposed lung sound classification system. Substantial variation was observed in the included studies in terms of study subjects, sample size, and use of feature extraction methods and sound classification models. For instance, seven types of feature extraction methods and nine types of sound classification models/algorithms were used across the studies. Hence, we could not draw any conclusion from this systematic review. However, the reported accuracies for classifying adventitious lung sounds ranged from 66.3% to 100%. This review identified only one study [21] that involved children aged under 5 years with pneumonia to evaluate an integrated computerized lung sound classification framework to detect adventitious lung sounds at an accuracy of 86.7% (sensitivity = 86.5%; specificity = 86. 6%). Although the rest of the studies demonstrated excellent accuracy, sensitivity, and specificity values, their results were primarily limited to overall diagnostic accuracy parameters in classifying lung sounds and did not provide disaggregated results by age and/by target condition/s. Therefore, the findings could not be directly applied to the paediatric population to diagnose childhood pneumonia. These shortfalls are important findings of this review and suggest a need for improved reporting of study findings. The authors should account for participants’ contributing factors (such as age, disease/target condition) while analysing and reporting their data. Evidently, the methodological quality of the included studies was deemed to be unclear due to insufficient reporting in participant selection, sampling methods, and clinical/target conditions of the participants. The concerns regarding the applicability of the patient selection and index test domain were unclear and high, respectively.

To the best of our knowledge, this is the first systematic review assembling evidence on the discriminatory power of digital auscultation for the detection of adventitious lung sound/s against conventional auscultation or acoustic analysis of recorded lung sounds by physicians in childhood pneumonia diagnosis. Prior reviews focused on summarizing existing evidence on artificial intelligence and algorithms to classify adventitious lung sounds [27,42]. Another systematic review and meta-analysis by Gurung et al. [20] evaluated the performance of computerized lung sounds analysis to detect adventitious lung sounds in respiratory diseases against chest radiography or clinical diagnosis.

In line with our findings, this review emphasised methodological and analytical standardization, including completeness and transparency in reporting by following standardized guidelines, and the need for conducting more studies on the paediatric population. To date, the Standards for Reporting of Diagnostic Accuracy Studies (STARD) 2015 statement remains the most used tool for reporting of studies investigating diagnostic test accuracy and performance [43,44]. However, this tool has some shortcomings when reporting studies evaluating artificial intelligence (AI) driven interventions due to unclear methodological interpretation, lack of standardised nomenclature, use of unfamiliar outcome measures, and other issues, thereby limiting the comprehensive appraisal of these technologies [45]. Thus, our study findings further reiterate the need for developing an AI-specific STARD guideline to ensure complete and robust reporting of studies evaluating AI-driven technologies and interventions [45,46].

The heterogeneous nature of the included studies and insufficient data prohibited us from performing meta-analyses and drawing conclusions on the diagnostic accuracy of digital auscultation and computerized analysis of lung sounds in childhood pneumonia diagnosis.

## CONCLUSIONS

Given the paucity of available data in this area, there is a need for additional well-designed studies to generate evidence on accuracy, sensitivity, and specificity of digital auscultation and/or computerized analysis of lung sounds involving the paediatric population to diagnose pneumonia. Future investigations should also consider conducting the studies in real-life, noisy clinical settings rather than in a controlled laboratory or clinical environment to broaden the usability of the automated applications. This is a rapidly evolving field, and new and advanced applications with robust reporting of methods and findings within studies will enable meta-analysis in future reviews.

## Additional material


Online Supplementary Document
