# Supplementary material for: Digital auscultation as a diagnostic aid to detect childhood pneumonia: A systematic review
Source: J Glob Health. 2022 Apr 23;12:04033. doi: 10.7189/jogh.12.04033 (PMC9024283; doi:10.7189/jogh.12.04033)
Supplement: Online Supplementary Document [file jogh-12-04033-s001.pdf]

Appendix S1: Search strategy for databases

Name of the database: Ovid MEDLINE(R) 1946 to October Week 5 2021

Search date: 27 October 2021

1	exp pneumonia/ or exp respiratory tract infections/	487441
2	(pneumon* or bronchpneumon* or pleuropneumon*).tw.	185517
3	(lower respiratory tract infection* or lower respiratory infection* or lrti).tw.	7651
4	("gram negative bacilli" or "pseudomonas aeruginosa" or "pseudomonas aruginosa" or enterobacter$ or pneumonitis or "pulmonary inflammation" or "lung inflammation").ti,ab.	109801
5	Or/1-4	672032
6	Respiratory Sounds/ or pulmonary sound*.mp. or respiratory sound*.mp.	9663
7	(analy* or detect* or auscultation).mp.	8821403
8	Auscultation/ or computer* auscultation.mp. or exp Signal Processing, Computer Assisted/ or exp Diagnosis, Computer-Assisted/ or Sound Spectrography/	155871
9	6 and (7 or 8)	3775
10	((digital* or electronic* or automat* or computer*) adj3 auscultation).tw.	138
11	((lung or respiratory or breath or pulmonary) adj3 sound* adj3 (analy* or detect* or auscultation)).mp.	446
12	Or/9-11	3988
13	((crackle* or wheez*) adj3 (detect* or analy* or auscultation)).tw.	459
14	(5 and 12) or 13	1211
15	limit 14 to ("newborn infant (birth to 1 month)" or "infant (1 to 23 months)" or "preschool child (2 to 5 years)")	606

Name of the database: Embase 1974 to 2021 Week 05

Search date: 27 October 2021

1	exp pneumonia/	336217
2	respiratory tract infections.mp. or exp respiratory tract infection/	427829
3	(pneumon* or bronchpneumon* or pleuropneumon*).tw.	284528
4	(lower respiratory tract infection* or lower respiratory infection* or lrti).tw.	12413
5	("gram negative bacilli" or "pseudomonas aeruginosa" or "pseudomonas aruginosa" or enterobacter$ or pneumonitis or "pulmonary inflammation" or "lung inflammation").ti,ab.	157595
6	Or/1-5	825633
7	exp abnormal respiratory sound/ or exp wheezing/ or pulmonary sound.mp.	66116
8	auscultation.mp. or auscultation/ or exp lung auscultation/	17687
9	7 and 8	4092
10	((digital* or electronic* or automat* or computer*) adj3 auscultation).tw.	193
11	((lung or respiratory or breath or pulmonary) adj3 sound* adj3 (analy*or detect* or auscultation)).mp.	817
12	or/9-11	4722
13	((crackle* or wheez*) adj3 (detect* or analy* or auscultation)).tw.	1087
14	(6 and 12) or 13	2538
15	exp Child/ or Child*.tw.	3130174
16	exp Infant/ or Infant*.tw.	1135822

17	(paediatric* or pediatric* or toddler* or preschool*).tw.	641319
18	(infant* or infancy or newborn* or baby* or babies or neonat* or preterm* or prematur*).tw.	1048785
19	Or/15-18	3616232
20	14 and 19	834

Name of database: Global Health 1973 to 2021 Week 05

Search date: 27 October 2021

1	pneumonia.mp. or exp pneumonia/	33494
2	exp lower respiratory tract infections/	10421
3	respiratory tract infections.mp.	9965
4	(pneumon* or bronchpneumon* or pleuropneumon*).tw.	58307
5	(lower respiratory tract infection* or lower respiratory infection* or lrti).tw.	5491
6	("gram negative bacilli" or "pseudomonas aeruginosa" or "pseudomonas aruginosa" or enterobacter$ or pneumonitis or "pulmonary inflammation" or "lung inflammation").ti,ab.	40785
7	or/1-6	100801
8	clinical examination/ or exp auscultation/	1624
9	((digital* or electronic* or automat* or computer*) adj3 auscultation).tw.	10
10	((lung or respiratory or breath or pulmonary) adj3 sound* adj3 analy*).mp.	16
11	or/8-10	1643
12	((crackle* or wheez*) adj3 (detect* or analy* or auscultation)).tw.	109
13	(7 and 11) or 12	188
14	child*.tw.	311973
15	Infant*.tw	100996
16	(paediatric* or pediatric* or toddler* or preschool*).tw.	62596
17	(infant* or infancy or newborn* or baby* or babies or neonat* or preterm* or prematur*).tw.	139379
18	or/14-17	394714
19	13 and 18	102

Name of database: CINAHL Plus

Search date: 27 October 2021

S1	(MH "Respiratory Tract Infections+")	89808
S2	(MH "Respiratory Syncytial Virus Infections")	2062
S3	TX LRTI	402
S4	S1 OR S2 OR S3	91122
S5	(MH "Signs and Symptoms, Respiratory+") OR (MH "Respiratory Sounds+")	32730
S6	TX respiratory sound	3995
S7	TX respiratory sound* OR TX pulmonary sound* OR TX lung sound* OR TX breath sound*	5258
S8	S5 OR S6 OR S7	34952
S9	(MH "Sound Spectrography")	794
S10	TX (analy* or detect* or auscultation)	2042380
S11	S9 OR S10	2042670

S12	S8 AND S11	10691
S13	TX auscultation N15 (digital* or electronic* or automat* or computer*)	127
S14	TX sound* N15 (analy* or detect* or auscultation) N15 (lung or respiratory or breath or pulmonary)	205
S15	S12 OR S13 OR S14	10786
S16	S4 AND S15	921
S17	TX (detect* or analy* or auscultation) N15 ((crackle* or wheez*)	479
S18	S16 OR S17	1332
S19	(MH "Child, Preschool") OR "children"	487369
S20	(MH "Infant+") OR "infant"	301860
S21	(MH "Infant, Newborn+") OR "neonate"	151306
S22	TX (paediatric* or pediatric* or toddler* or preschool*)	659202
S23	TX (infant* or infancy or newborn* or baby* or babies or neonat* or preterm* or prematur*)	588491
S24	S19 OR S20 OR S21 OR S22 OR S23	1048783
S25	S18 AND S24	142

Name of database: Scopus

Search date: 27 October 2021

(((ALL(respiratory tract infection)) OR (ALL(lower respiratory infection)) OR (ALL(lrti)) OR (ALL(pneumon* OR bronchpneumon* OR pleuropneumon*)) OR (TITLE-ABS-KEY("gram negative bacilli" or "pseudomonas aeruginosa" or "pseudomonas aruginosa" or enterobacter$ or pneumonitis or "pulmonary inflammation" or "lung inflammation"))) AND ((ALL(auscultation W/15 digital* OR electronic* OR automat* OR computer*)) OR (ALL(analy* W/15 "respiratory sound" OR "pulmonary sound" OR "lung sound" OR "breath sound")))) OR (ALL((detect* OR analy* OR auscultation) W/3 (crackle* OR wheez*)))) AND ((TITLE-ABS-KEY(paediatric* OR pediatric* OR toddler* OR preschool*)) OR (TITLE-ABS-KEY(child* OR infant*)) OR (TITLE-ABS-KEY(infant* or infancy or newborn* or baby* or babies or neonat* or preterm* or prematur*)))	1345

Name of database: Web of science

<u>Web of Science Core Collection</u> (1900-present): (Science Citation Index Expanded: 1900-present; Scocial Science Citation Index: 1900-present; Emerging Sources Citation Index: 2015-present)

Search date: 27 October 2021

1	**TOPIC:** (lower respiratory infection)	33453
2	**TOPIC:** (respiratory tract infection)	43076
3	**TOPIC:** (pneumon* OR bronchpneumon* OR pleuropneumon*)	246000
4	**TOPIC:** ("gram negative bacilli" or "pseudomonas aeruginosa" or "pseudomonas aruginosa" or enterobacter$ or pneumonitis or "pulmonary inflammation" or "lung inflammation")	141884
5	#4 OR #3 OR #2 OR #1	393725
6	**TOPIC:** ((lung or respiratory or breath or pulmonary) sound*)	6160
7	**TOPIC:** (analy* OR detect* OR auscultation)	16,924,176
8	#7 AND #6	3235

9	**TOPIC:** ((digital* or electronic* or automat* or computer*) NEAR/3 auscultation)	274
10	#9 OR #8	3415
11	#10 AND #5	338
12	**TOPIC:** ((crackle* OR wheez*) NEAR/3 (detect* OR analy* OR auscultation))	652
13	#12 OR #11	957
14	**TOPIC:** (child* OR infant*)	2383,798
15	**TOPIC:** (paediatric* or pediatric* or toddler* or preschool*)	532166
16	**TOPIC:** (newborn* OR baby* OR babies OR neonat* OR preterm* OR prematur*)	665218
17	#16 OR #15 OR #14	2993355
18	#17 AND #13	398

Name of database: IEEEXplore

Search date: 27 October 2021

((crackle* OR wheez* OR lung sound OR respiratory sound OR breath sound OR pulmonary sound) NEAR/3 (detect* OR analy* OR auscultation OR digital OR electronic OR automated OR computer*))	338

Name of database: Clinicaltrial.gov

Search date: 27 October 2021

(lung sound OR respiratory sound OR pulmonary sound OR breath sound OR crackle OR wheeze) AND (detect OR analysis OR diagnose OR auscultation OR digital OR electronic OR automated OR computer)	33

Appendix S2: Excluded studies with reasons for exclusion

Sl	Authors, Year	Title	Reason for exclusion
1	Scrafford et al., 2016	Evaluation of digital auscultation to diagnose pneumonia in children 2 to 35 months of age in a clinical setting in Kathmandu, Nepal: a prospective case-control study	Reference test not used
2	Abeyratne, Swarnkar, Triasih, & Setyati, 2013	Cough sound analysis - a new tool for diagnosing pneumonia	Index test not used
3	Ellington et al., 2012	Computerised lung sound analysis to improve the specificity of paediatric pneumonia diagnosis in resource-poor settings: protocol and methods for an observational study	Study protocol
4	Elphick et al., 2004	Validity and reliability of acoustic analysis of respiratory sounds in infants	Results could not be stratified by disease condition
5	Fischer et al., 2016	Relationship Between Computerized Wheeze Detection and Lung Function Parameters in Young Infants	Index test not used
6	Furman, Malinin, Furman, & Sokolovsky, 2014	Computer-Assisted Assay of respiratory sounds of Children suffering from Bronchial Asthma	Target condition not matched
7	Gross et al., 2007	Mobile nocturnal long-term monitoring of wheezing and cough	Results could not be stratified by disease condition
8	Guntupalli, Alapat, Bandi, & Kushnir, 2008	Validation of Automatic Wheeze Detection in Patients with Obstructed Airways and in Healthy Subjects	Study population and target condition not matched
9	Haider, Joseph, & Periyasamy, 2017	An investigation on the statistical significance of spectral signatures of lung sounds	Reference test not used
10	Lozano, Fiz, & Jané, 2016	Automatic Differentiation of Normal and Continuous Adventitious Respiratory Sounds Using Ensemble Empirical Mode Decomposition and Instantaneous Frequency	Reference test not used
11	Kang, Karpate, Almulla, Teach, & Shekhar, 2016	Automatic Identification of Wheezing in Auscultated Lung Sounds	Conference abstract
12	Kosasih, Abeyratne, Swarnkar, & Triasih, 2015	Wavelet Augmented Cough Analysis for Rapid Childhood Pneumonia Diagnosis	Index test not used
13	Julian et al., 2017	Automatic crackle detection in children with pneumonia	Conference abstract
14	Lovrenski, 2016	Stethoscope vs. ultrasound probe - which is more reliable in children with suspected pneumonia?	Index test not used
15	McCollum et al., 2017	Listening panel agreement and characteristics of lung sounds digitally recorded from children aged 1–59 months enrolled in the Pneumonia Etiology Research for Child Health(PERCH) case–control study	Index test not used

Sl	Authors, Year	Title	Reason for exclusion
16	Emmanouilidou et al., 2017	Digitally Recorded Lung Sounds And Mortality Among Children 1-59 Months Old With Pneumonia In The Pneumonia Etiology Research For Child Health Study	Index test not used
17	Oletic & Bilas, 2018	Asthmatic Wheeze Detection From Compressively Sensed Respiratory Sound Spectra	Target condition not matched
18	Pasterkamp, Wiebicke, & Fenton, 1987	Subjective assessment vs computer analysis of wheezing in asthma	Target condition not matched
19	Pingale & Patil, 2017	Analysis of Cough Sound for Pneumonia Detection Using Wavelet Transform and Statistical Parameters	Lung sounds were not used
20	Prodhan et al., 2008	Wheeze Detection in the Pediatric Intensive Care Unit: Comparison Among Physician, Nurses, Respiratory Therapists, and a Computerized Respiratory Sound Monitor	Results could not be stratified by disease condition
21	Puder et al., 2014	Validation of computerized wheeze detection in young infants during the first months of life	Target condition not matched
22	(Puder, Wilitzki, Bührer, Fischer, & Schmalisch, 2016)	Computerized wheeze detection in young infants: comparison of signals from tracheal and chest wall sensors	Target condition not matched
23	Qiu, Whittaker, Lucas, & Anderson, 2005	Automatic wheeze detection based on auditory modelling	Unclear subjects' age and target condition
24	Song, 2015	Diagnosis of Pneumonia From Sounds Collected Using Low Cost Cell Phones	Reference test not used
25	Ahmed, McCollum, Nair, Cunningham, & Baqui, 2020	Community use of digital auscultation to improve diagnosis of childhood pneumonia in low resource setting	Conference abstract
26	Dramburg, Dellbrügger, van Aalderen, & Matricardi, 2021	The impact of a digital wheeze detector on parental disease management of pre-school children suffering from wheezing—a pilot study	Target condition not matched
27	Rennoll, McLane, Emmanouilidou, West, & Elhilali, 2021	Electronic Stethoscope Filtering Mimics the Perceived Sound Characteristics of Acoustic Stethoscope	Index test not used
28	Ferreira-Cardoso et al., 2021	Lung auscultation using the smartphone—feasibility study in real-world clinical practice	Index test not used
29	Bertrand, Segall, Sánchez, & Bertrand, 2020	Lung auscultation in the 21th century	Review article
30	Habukawa et al., 2021	Wheeze recognition algorithm for remote medical care device in children: Validation study	Target condition not matched
31	Khan, Badashah, & Mudda, 2019	Preliminary detection of lung diseases in pediatric population using soft computing	Reference test not used; unclear subject's disease condition

Sl	Authors, Year	Title	Reason for exclusion
32	Kotb et al., 2020	The machine learned stethoscope provides accurate operator independent diagnosis of chest disease	Reference test not used
33	Ramanathan et al., 2019	Digital stethoscopes in paediatric medicine	Review article
34	Rocha et al., 2019	An open access database for the evaluation of respiratory sound classification algorithms	Index test not used
35	Khan, Jawarkar, & Ahmed, 2012	Cell phone based Remote Early Detection of Respiratory Disorders for Rural Children using Modified Stethoscope	Target condition not matched
36	Murphy et al., 2004	Automated lung sound analysis in patients with pneumonia	Reference test was not used

Appendix S3: Data extraction form

**Reviewer**	
**Date form completed**	

A. Study details

**Study ID**	
**Title**	
**Author**	
**Year Published**	
**Journal**	
**Location** (country/city)	

B. Study design/method

		Location in text (page/ figure/ table/ other)
**Type of study**	Randomised controlled trial……………….☐<br>Cross-sectional study…………………….☐<br>Other ……………………………….	
**Study Setting**	Hospital:….……………………………….☐<br>Community:……………..……………….☐<br>Other: ……………………………….	
**Study period**		
**Sampling method**		
**Sample size**		

C. Population characteristics

		Location in text (page/ figure/ table/ other)
**Target condition**	Pneumonia:……………………….……..☐<br>ALRI:...……………………………………..☐<br>Asthma………………………..……….☐<br>Other: ……………………………….	
**Enrolment Eligibility<br>A. inclusion Criteria<br>B. Exclusion Criteria**		
**Case definition**		
**Total number enrolled**		
**Age range (mean/median age)**		
**Gender (% female)**		
**Total number included in the analysis**		
**Notes**		

D. Index test/s

		Location in text (page/ figure/ table/ other)
**Recording device**		
**Brand of stethoscope**		
**Detail description of the index test/s**		
**Sound analysis software/technology used**		
**Lung sound classified** (crackles, wheezes, pleural rub etc.)		
**Notes**		

E. Reference test

		Location in text (page/ figure/ table/ other)
**Type of acoustic analysis**	Conventional auscultation ……………..☐<br>Analysis of recorded lung sounds …..☐	
**Brand of stethoscope/recording device**		
**Detail description of reference test**		
**Lung sound classified** (crackles, wheezes, pleural rub etc.)		
**Notes**		

F. Study findings

		Location in text (page/ figure/ table/ other)
True positive		
False positive		
False negative		
True negative		
Sensitivity (95% CI)		
Specificity (95% CI)		
Positive predictive value (95% CI)		
Negative predictive value (95% CI)		
Accuracy (95% CI)		
Kappa value		
**Notes**		
